# Cytokine dynamics in the blood and cerebrospinal fluid of HIV/AIDS patients with cryptococcal meningitis receiving antifungal therapy

**DOI:** 10.3389/fcimb.2025.1691025

**Published:** 2025-12-05

**Authors:** Rui Su, Shuwei Zhao, Jun Liu, Chongxi Li, Yuye Li, Yingkui Cao, Zhenghui Yang, Hongbin Li

**Affiliations:** 1 Department of Dermatology and Venereology, First Affiliated Hospital of Kunming Medical University, Kunming, China; 2 Department of Acquired Immunodeficiency Syndrome (AIDS)/Sexually Transmitted Disease (STD), The Third People’s Hospital of Kunming, Yunnan, China

**Keywords:** AIDS, *cryptococcus*, antifungal therapy, cytokines, cerebrospinal fluid

## Abstract

**Background:**

Cytokines in the serum and cerebrospinal fluid (CSF) are critical to the pathogenesis of HIV-associated cryptococcal meningitis (HCM). Previous studies focused on baseline cytokine levels, but changes during antifungal therapy are underexplored.

**Methods:**

Twenty-three patients with a first episode of HCM were prospectively followed to evaluate immune cell and cytokine dynamics during antifungal treatment therapy. Clinical features and laboratory data were systematically collected, and principal component and correlation analyses were performed to identify immune factors associated with HCM.

**Results:**

Typical clinical manifestations include fever, headache, and nausea, among others. All patients presented severely low CD4^+^ T-cell counts and a notable reduction in CD8^+^ T cells. Over 28 days of antifungal treatment, significant decreases in the CSF levels of IL-2 and IL-10 and the plasma levels of IL-10 and IL-4 were observed. The plasma TNF-α concentration remained stable from days 1 to 14, followed by a marked increase on day 21. Plasma IFN-γ was negatively correlated with neutrophils (P = 0.04, r = -0.297), while IL-1β was positively correlated with leukocytes (P = 0.0031, r = 0.4266), and IL-8 was negatively correlated with lymphocytes (P = 0.0074, r = 0.3901). No significant correlations were found between other factors and neutrophils, leukocytes, or lymphocytes (P > 0.05).

**Conclusion:**

Cytokines derived from innate immune cells play a central role in host defense against HIV-associated cryptococcal infections. The observed reductions in cytokine levels in both plasma and CSF after antifungal therapy provide new insights into the immune response in HCM, highlighting the dynamic interplay of immune factors during treatment.

## Introduction

Meningoencephalitis caused by *Cryptococcus neoformans* has a high mortality rate and substantial incidence, particularly among immunocompromised individuals, with a significant impact on HIV-infected patients. Despite the widespread use of antiretroviral therapy (ART) and antifungal medications in the past decade, which have aided in managing HIV-related cryptococcal meningitis and decreasing its global incidence, the mortality rate remains alarmingly high. Approximately 50% of AIDS patients with cryptococcal meningitis still succumb to infection, especially in medical resource-limited settings (Tugume et al., 2023).

HIV-associated cryptococcal meningitis typically has an insidious onset and atypical clinical symptoms, which complicates timely diagnosis and treatment. The relatively low treatment success rate and high relapse tendency have long been challenging in clinical practice (Williamson, 2017). Currently, the development of novel antifungal drugs has made limited progress, and fundamentally overcoming the treatment bottleneck is difficult. In the process of exploring more effective treatment strategies, the crucial factors affecting treatment response and clinical outcomes in cryptococcosis patients are increasingly recognized to be intimately tied to the host immune response. At present, the underlying mechanisms involved have not been fully elucidated. However, precisely against this backdrop, cryptococcal immunotherapy, which aims to modulate the immune response and improve prognosis, has attracted extensive attention (Antachopoulos and Walsh, 2012; Tenforde et al., 2017; Williamson et al., 2017). An increasing body of evidence continuously emphasizes that the host immune response to cryptococcosis plays a crucial role in determining disease manifestations and outcomes (Meya et al., 2016; Neal et al., 2017; Pirofski and Casadevall, 2017).

However, most of our understanding of the immune response to cryptococcal infection is based on *in vitro* experiments and animal studies. Research in animal models suggests that type 1 helper T (Th1) cell responses are protective, whereas type 2 helper T (Th2) cell responses are generally nonprotective or even detrimental (Wormley et al., 2007; Wozniak et al., 2009; Muller et al., 2013). In contrast, the limited data available from human studies indicate a more complex pattern of effector T-cell responses, differing from those observed in animal models (Jarvis et al., 2015; Scriven et al., 2017). Studies have demonstrated that elevated serum levels of IL-4 and IL-17 prior to ART can predict the subsequent development of immune reconstitution inflammatory syndrome (IRIS) in HCM patients (Boulware et al., 2010). Conversely, a study revealed that reduced IL-4 and IL-17 levels during IRIS were linked to high intracranial pressure (ICP) (Zheng et al., 2014). These paradoxical findings underscore the need for further investigations into the serum cytokines themselves. Furthermore, evidence indicates that cytokine levels in CSF do not always reflect those in serum, suggesting that local factors may play a critical role in central nervous system (CNS) diseases (Yoshio et al., 2016; Rodriguez-Smith et al., 2017).

Early studies focused primarily on baseline cytokine levels, with few reports addressing the dynamic variations in cytokines in plasma and CSF following antifungal therapy. In this study, by monitoring the dynamic changes in cytokines in CSF and plasma during antifungal treatment, we further revealed the immune response patterns associated with HIV-associated cryptococcal meningitis. This provides a certain basis for optimizing the treatment time window.

## Methods

### Ethical approval

All participant samples were collected under protocols approved by the institutional review board, with demographic and clinical data carefully documented on standardized forms. The Ethics Committee of the First Affiliated Hospital of Kunming Medical University approved this study, as evidenced by the approval document (2022 L-190), following the standard ethical review process. In accordance with the Declaration of Helsinki, all participants provided written informed consent.

### Study subjects

This study enrolled a cohort of 23 HIV-positive patients who experienced their first episode of cryptococcal meningitis between April 2022 and April 2024. Each patient met at least one primary diagnostic criterion: a positive cryptococcal smear in CSF, a positive India ink smear test of CSF, or a positive cryptococcal culture from CSF. In cases where initial test results were inconclusive, metagenomic next-generation sequencing (mNGS) of the CSF was used as a supplementary diagnostic tool. Blood samples were collected on days 1, 7, 14, 21, and 28 after antifungal treatment and postadmission. CSF collection was performed as clinically indicated by the attending physician on days 1, 7, 14 and 28 after therapy initiation. Upon admission, all participants underwent a detailed physical examination and a comprehensive set of laboratory tests, followed by antifungal treatment after diagnosis confirmation.

### Clinical data collection

All HIV patients with cryptococcal meningitis were clinically monitored, with comprehensive data collected on their demographics, disease characteristics, intracranial pressure, CSF biochemistry, routine laboratory results, treatments administered, and outcomes. Blood samples were collected on days 1, 7, 14, 21, and 28 after antifungal treatment and postadmission. CSF collection was performed on days 1, 7, 14 and 28 after therapy initiation. Blood samples were collected in EDTA anticoagulant tubes, followed by plasma and blood cell separation via centrifugation at 3500 rpm for 10 minutes. CSF samples were collected via standard tubes, and the supernatants were separated via centrifugation at 2500 rpm for 10 minutes. All plasma and CSF supernatant samples were immediately frozen at -80°C after collection for subsequent cytokine and chemokine analysis.

### ART treatment time and plan

Most of the 23 patients included in this study could not trace the initiation of antiretroviral therapy (ART) because of a complicated course or incomplete medical history. This limitation may stem from bias in patient memory of early treatment or omission of handover of medical history at referral to primary care facilities. However, all patients followed the HIV treatment regimen recommended by the World Health Organization (WHO), which uses a combination of Dolutegravir(s, DTG):


1. DTG + TAF/FTC (tenofovir amine/emtricitabine)

2. DTG + TDF/3TC (tenofovir fumarate/lamivudine)

### Evaluation and exclusion criteria for IRIS

IRIS diagnostic criteria: According to the international consensus, IRIS was defined as worsening after ART initiation (usually 3 months) and meeting the following conditions: (1) a decrease in the viral load by 1 log10 copies/mL or an increase in the CD4 T-cell count; (2) Exclude the aggravation of clinical symptoms caused by other infections, adverse drug reactions or treatment failure; (3) Pathological test results (such as the cryptococcal antigen titer) did not progress or decrease compared with those at baseline. IRIS monitoring method: All patients received regular clinical assessments (weekly neurological symptoms, body temperature, CSF pressure monitoring) and laboratory tests (plasma HIV viral load, CD4+ T-cell count, dynamic changes in CSF cryptococcal antigen titer) after enrollment to exclude potential IRIS events.

### Cytokine measurement

All plasma and cerebrospinal fluid (CSF) samples were tested for the following cytokines via the Luminex chip: IL-2, IL-4, IL-6, IL-8, IL-10, IL-13, IL-1β, IL-12P70, IFN-γ, and TNF-α.

### Statistical analyses

Clinical data are presented as case numbers (percentages), means ( $\bar{\text{x}}$) with standard deviations, or medians with interquartile ranges. Statistical analyses were performed via the t test and Mann–Whitney U test for continuous variables and Fisher’s exact test for categorical variables. Cytokines and chemokines in both CSF and plasma were analyzed via principal component analysis (PCA) to identify linearly independent principal components (PCs) through dimensionality reduction. The associations between these PCs and various cytokines/chemokines were examined. Variables with a P value < 0.05 in the univariate analysis were included in the multivariate analysis, with outcomes reported as odds ratios (ORs) and their corresponding 95% confidence intervals (CIs). Data analysis was conducted via SPSS 26.0 software (SPSS Inc., Chicago, IL). Graphical representations were created with GraphPad Prism 10 (GraphPad Software, San Diego, CA) and Origin Pro software (OriginLab Corporation, Northampton, MA).

## Results

### Demographic and clinical profiles of individuals with HIV-related cryptococcal meningitis

Over a two-year period, a total of 23 cases of AIDS-associated cryptococcal meningitis were documented. In all patients, metagenomic next-generation sequencing (mNGS) of the cerebrospinal fluid (CSF) identified the pathogen as *Cryptococcus neoformans*. Among these patients, 19 (82.6%) patients survived, whereas 4 (17.4%) unfortunately passed away. The average age of the deceased group was significantly greater than that of the survivor group, with mean ages of 62.25 and 45.26 years, respectively (P = 0.012). The most common clinical manifestations in patients with cryptococcal meningitis include fever, headache, nausea, vomiting, cough, and weight loss. A subset of patients also exhibit neurological symptoms, such as altered consciousness, seizures, blurred vision, pathological reflexes, and signs of meningeal irritation. However, no statistically significant differences were observed in the clinical symptoms between the deceased and surviving patients. A significant difference was found in the baseline opening pressure of the CSF between the two groups (P = 0.038). The deceased group generally presented a lower opening pressure, often below 100 mmHg, whereas the survivors presented a CSF opening pressure exceeding 200 mmHg.

Laboratory tests conducted on the 23 infected patients revealed a significantly greater neutrophil count in the blood of the surviving group than in that of the deceased group (3.94 vs. 2.18, P = 0.033). A decrease in CD4 and CD8 levels was observed in all 23 patients; however, no statistically significant difference was found between the two groups. The primary therapeutic regimen for both groups was amphotericin B combined with fluconazole, with 5-fluorocytosine added as a supplemental measure in some cases to reduce acute mortality (**Table 1**).

**Table 1:** Baseline characteristics of patients with HIV-associated cryptococcal meningitis, stratified by survival outcome.

	Total case (n=23)		p
	Survival (n=19,82.6%)	Death (n=4,17.4%)	
**Age** (years)	45.26±10.91	62.25±13.12	.012*
Clinical symptoms			
Fever	10(76.9%)	3(23.1%)	.604
Dizziness and headache	15(88.2%)	2(11.8%)	.270
Cough and sputum	6(75%)	2(25%)	.490
Nausea and vomiting	6(85.7%)	1(14.3%)	.792
Lose weight	7(87.5%)	1(12.5%)	.644
Neurological symptoms and signs			
Disturbance of consciousness	7(100%)	0	.273
Convulsion	2(66.7%)	1(33.1%)	.453
Blurred vision	8(100%)	0	.257
Psychobehavioral abnormality	2(100%)	0	.999
Swoon	1(50%)	1(50%)	.324
Decreased muscle strength	2(66.7%)	1(33.1%)	.453
Memory decline	4(100%)	0	.999
Meningeal irritation sign	13(92.9%)	1(7.1%)	.260
Pathological sign	6(100%)	0	.539
**Intracranial pressure**			.038*
>200 mmHg	16(88.9%)	2(11.1%)	
200 mmHg∼100 mmHg	3(100%)	0	
≦100 mmHg	0	2(100%)	
Laboratory examination			
Leukocyte in blood(*10^9/l)	5.28(2.92,1.35∼15.46)	3.22(3.03,1.25∼4.46)	.081
**Neutrophils in blood** (*10^9/l)	3.94(1.65,0.72∼12.94)	2.18(1.34,1.28∼2.79)	.033*
Lymphocyte in blood(*10^9/l)	0.54(0.47,0.21∼3.91)	0.82(1.48,0.35∼2.03)	.837
Monocyte in blood(*10^9/l)	0.42(0.47,0.09∼0.8)	0.3(0.3,0.19∼0.52)	.534
CD4	34.1(34.94,5.62~211)	55.91(70.1,25.6~115.46)	.227
<50 /ul	12(85.7%)	2(14.3%)	
50-200/ul	5(71.4%)	2(28.6%)	
>200 /ul	2(100%)	0	
CD8(/ul)	344.5(274.9,134~3614)	777(1289.58,169~1565.77)	.538
RNA	2.44E+5(2.9E+6,100~6.47E+6)	8.38E+5(5.78E+6,4.45E+5~8E+6)	.250
Medicate			.247
Amphotericin B+Fluconazole+Fluorocytosine	10(90.9%)	1(9.1%)	
Amphotericin B+Fluconazole	7(77.8%)	2(22.2%)	
AmphotericinB+Fluorocytosine	2(100%)	0	
Amphotericin B+Fluiconazole	0	1(100%)	

*Statistically significant.

The Fisher-Freeman-Halton exact test for CD4 cell count and prognosis of 23 included patients with HIV-related cryptococcal meningitis revealed that the distribution of CD4 counts showed a significant immunosuppression state: 60.9% (14/23) of patients with CD4 <50 cells/µL, 30.4% (7/23) at 50–200 cells/µL and only 8.7% (2/23) >200 cells/µL. The incidence of poor prognosis was 17.4% (4/23), with 50% (2/4) in the CD4 <50 cells/µL group and 50% (2/4) in the CD4 50–200 cells/µL group. No adverse prognosis was observed in the CD4 >200 cells/μL group. Preliminary trends suggest that severe immunosuppression (CD4 <50 cells/µL) may be associated with a poor outcome, but this finding was verified with a larger sample. The proportion of patients with a poor prognosis in the CD4 <50 cells/µL group was lower than that in the CD4 50–200 cells/µL group, but the difference was not significant (P > 0.5).

The HIV RNA load was lower in surviving patients than in deceased patients (P > 0.5). The HIV RNA load was negatively correlated with the CD4/CD8 ratio (r = -0.2876, p = 0.2326) and with the CD4 count (r = -0.1452, p = 0.5531). Compared to day 1 of antifungal therapy, the HIV RNA load decreased on days 21, 28, and 35 (P > 0.5).

### IRIS occurrence

Among the 23 patients, no patients meeting the above IRIS diagnostic criteria were observed during the ART treatment period (until 28 days to the follow-up endpoint).

### Cerebrospinal fluid and plasma cytokine levels over a 28-day period in patients with cryptococcal meningitis

**Figure 1:**
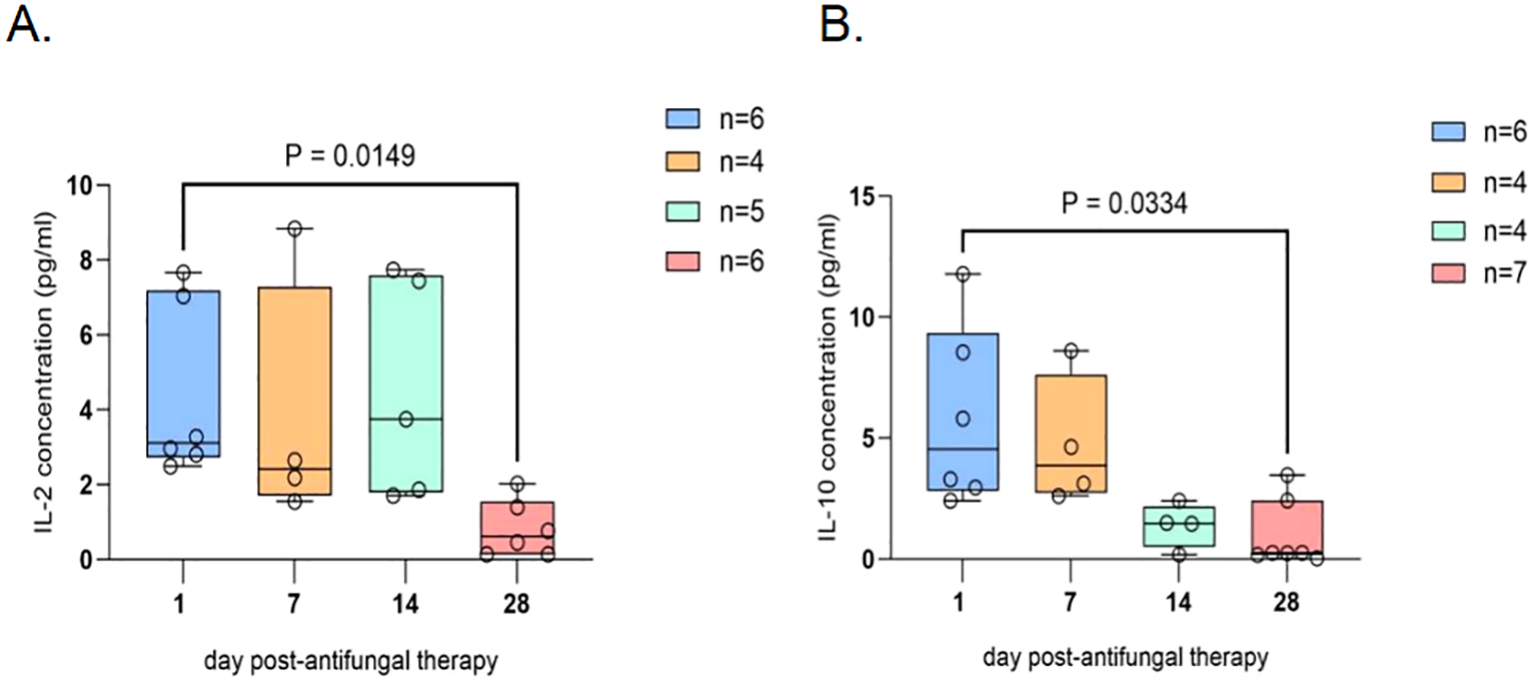
Cytokine trends in the CSF of patients with HIV-associated cryptococcal meningitis. CSF samples from 23 cryptococcal meningitis patients on days 1, 7, 14, and 28 were meticulously selected for cytokine detection and the subsequent construction of boxplots. where “n” represents the sample size of the included statistics. **(A)** Elucidating the dynamic evolution of IL-2 in CSF after antifungal therapy: A pronounced decline in IL-2 levels within the CSF was observed on day 28 after antifungal treatment. **(B)** Tracking the dynamic alterations in IL-10 in CSF following antifungal agent administration: A statistically significant reduction in the IL-10 concentration within the CSF was noted on day 28 after the initiation of antifungal therapy. Sample loss occurred due to logistical challenges during sample collection and processing. Additionally, extreme outliers were excluded from the analysis to ensure data reliability. The exclusion criteria for outliers were defined as values exceeding [X] standard deviations from the mean. The final analysis included meticulously selected samples that met the quality control criteria, and the sample size for each time point is explicitly labeled on the graph.

**Figure 2:**
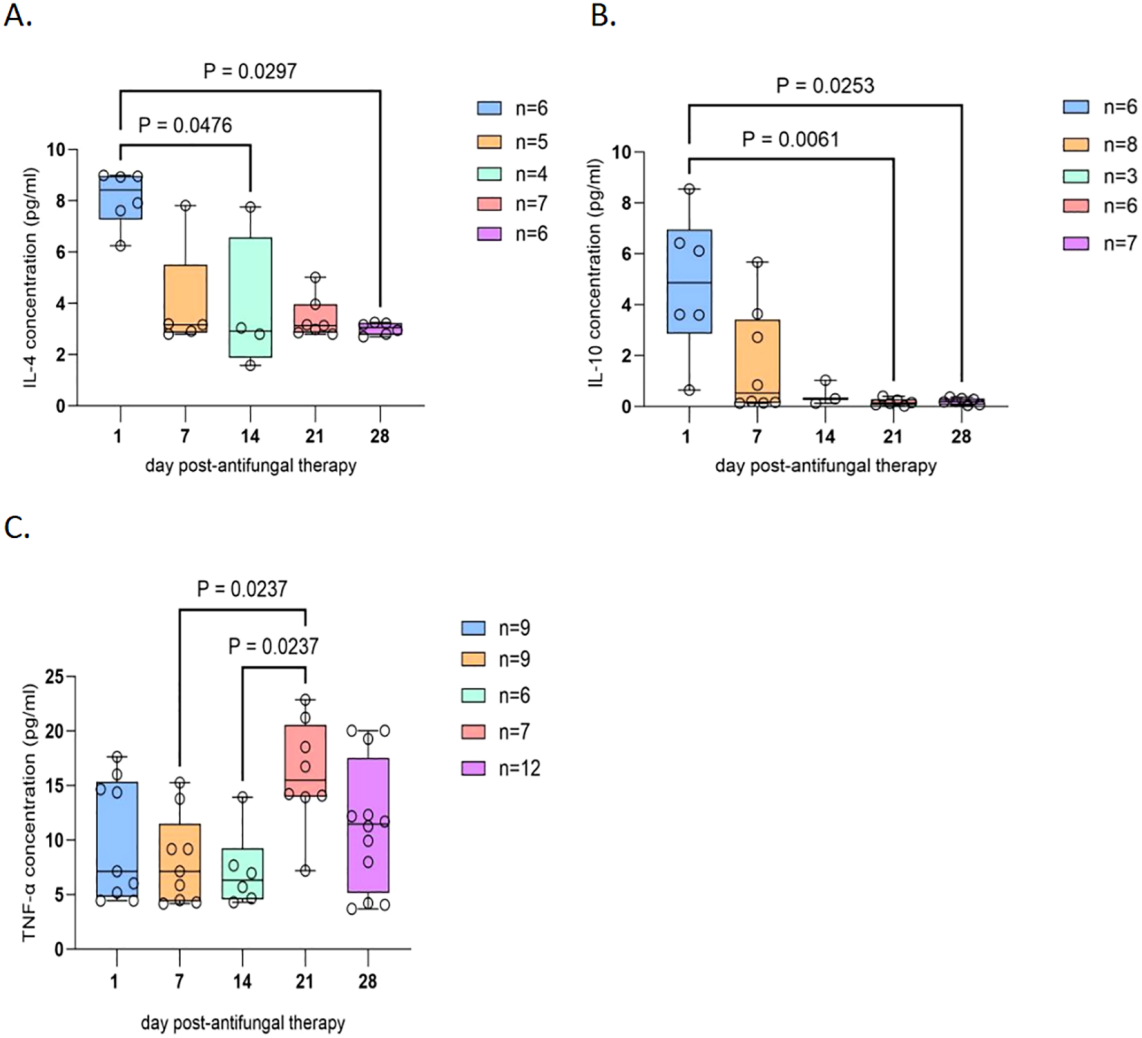
Cytokine trends in the plasma of patients with HIV-associated cryptococcal meningitis. Plasma samples from 23 enrolled cryptococcal meningitis patients on days 1, 7, 14, 21, and 28 were chosen for cytokine detection and boxplot analysis. where “n” represents the sample size of the included statistics. **(A)** Dynamic changes in plasma IL-4 following antifungal treatment revealed a notable decreasing trend at days 14 and 28 posttreatment. **(B)** Dynamics of plasma IL-10 over 28 days after antifungal therapy indicate a significant decreasing trend on days 21 and 28 after treatment. **(C)** Plasma dynamic TNF-α levels over 28 days after antifungal therapy. The level of TNF-α was significantly greater at 21 days than at 7 and 14 days. Sample loss occurred due to logistical challenges during sample collection and processing. Additionally, extreme outliers were excluded from the analysis to ensure data reliability. The exclusion criteria for outliers were defined as values exceeding [X] standard deviations from the mean. The final analysis included meticulously selected samples that met the quality control criteria, and the sample size for each time point is explicitly labeled on the graph.

Boxplots were generated on the basis of data collected over 28 days from patients with cryptococcal meningitis. In CSF, a significant decrease in the levels of IL-2 and IL-10 was observed over the four-week period, with statistically significant differences noted between baseline and day 28 (P = 0.0149 and P = 0.0334, respectively) (**Figure 1**). Similarly, in the plasma, significant reductions in the levels of IL-4 and IL-10 were observed. IL-4 was significantly lower on days 14 and 28 than at baseline, with P values of 0.0476 and 0.0297, respectively. IL-10 levels also significantly decreased on days 21 and 28 compared with those at baseline, with P values of 0.0061 and 0.0253, respectively. Conversely, TNF-α was significantly greater on day 21 than on days 7 and 14 (P = 0.0237) (**Figure 2**).

Discrepancies in cytokine levels were observed between plasma and CSF. While the anti-inflammatory cytokine IL-10 was significantly decreased in both the plasma and CSF (P < 0.05), the level of IL-4 was significantly reduced only in the plasma (P < 0.05). For inflammatory cytokines, IL-2 was significantly decreased in the CSF (P < 0.05) but not in the plasma. The level of TNF-α increased significantly on day 21 in the plasma (P < 0.05) but not in the CSF.

### Cytokine correlation via PCA

**Figure 3:**
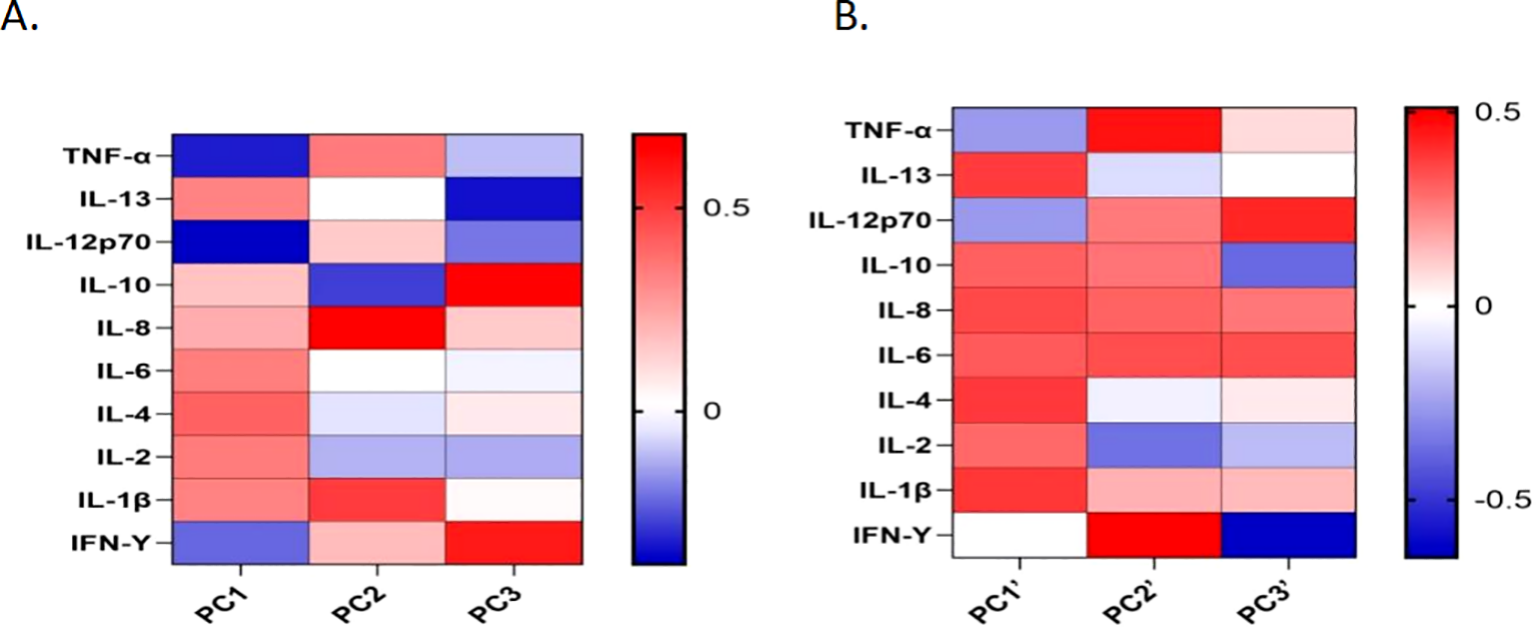
Cytokine principal component analysis (PCA) of plasma or CSF samples represented as a heatmap. **(A)** PCA of plasma samples revealed that PC1 is influenced primarily by IL-4, IL-12P70, IL-2, IL-6, IL-1β, IL-13, and TNF-α, with positive contributions from IL-4, IL-2, IL-6, and IL-1β, whereas IL-12P70 and TNF-α contribute negatively. PC2 is influenced mainly by IL-8, IL-1β, TNF-α (positive drivers), and IL-10 (negative drivers). **(B)** PCA loadings in CSF indicate that PC1’ is influenced primarily by IL-1β, IL-4, IL-13, and IL-8, all of which contribute positively. PC2’ is driven by IFN-γ, TNF-α, and IL-6 (positive contributions), with IL-2 contributing negatively. PC3’ is shaped mainly by IFN-γ, IL-12P70, IL-10, and IL-6, where IFN-γ and IL-10 contribute negatively, and IL-12P70 and IL-6 contribute positively.

Principal component analysis (PCA) was performed to assess cytokine correlations in plasma and CSF. For plasma, PCA revealed three principal components (PC1, PC2, and PC3) that together explained 76.09% of the total variation (**Figure 3**). For CSF, PCA revealed three components (PC1’, PC2’, PC3’) accounting for 86.63% of the variation (**Figure 3**).

**Figure 4:**
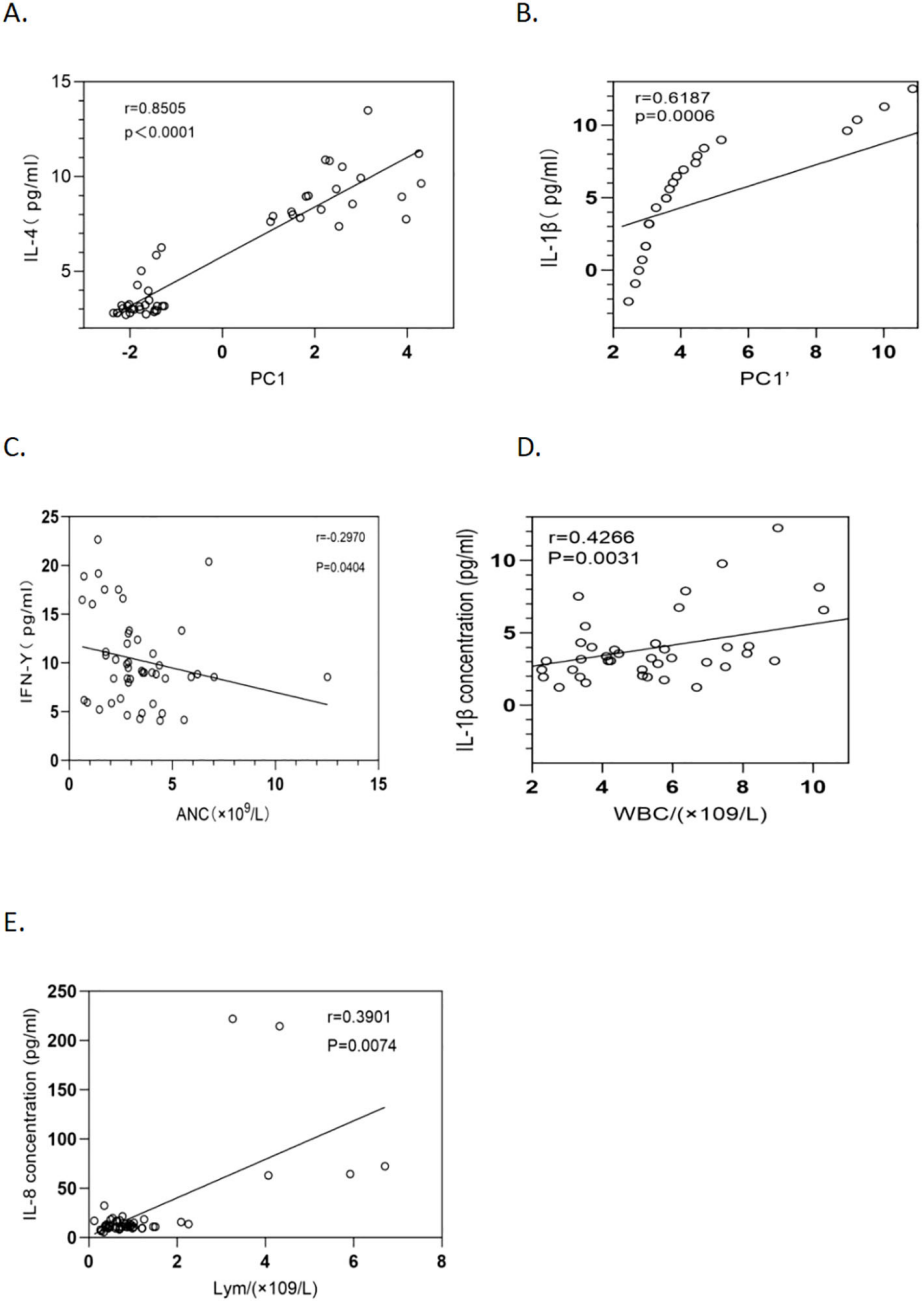
Relationships between cytokine concentrations and inflammatory cells, PC1, and PC1’ in 23 HCM patients. **(A)** The relationship between plasma IL-4 levels and PC1 over a 28-day period after antifungal therapy (P <0.0001, r = 0.8505) is shown with a fitted regression line and 95% confidence intervals. **(B)** The correlation between IL-1β levels in cerebrospinal fluid (CSF) and PC1’ over a 28-day period after antifungal therapy (P = 0.0006, r = 0.6187) is depicted with a regression line and 95% confidence intervals. **(C)** The relationship between the absolute neutrophil count and the IFN-γ level in HCM patients (P = 0.0404, r = -0.2970) is depicted with a regression line and 95% confidence intervals. **(D)** The association between the leukocyte count and the IL-1β level in HCM patients (P = 0.0031, r = 0.4266) is illustrated with a regression line and 95% confidence intervals. **(E)** The correlation between the lymphocyte count and the IL-8 level in HCM patients was thoroughly analyzed via an optimally fitted regression line with 95% confidence intervals (P = 0.0074, r = 0.3901).

PC1 was influenced by IL-4, IL-12P70, IL-2, IL-6, IL-1β, IL-13, and TNF-α, with IL-4, IL-2, IL-6, and IL-1β contributing positively and IL-12P70 and TNF-α contributing negatively. PC2 cells are driven mainly by IL-8, IL-1β, TNF-α, and IL-10. In the CSF, PC1’ was dominated by IL-1β, IL-4, IL-13, and IL-8, whereas PC2’ was influenced by IFN-γ, TNF-α, IL-2, and IL-6. PC3’ was affected by IFN-γ, IL-12P70, IL-10, and IL-6. PC1 was significantly positively correlated with IL-4 (P < 0.001, r = 0.8505) (**Figure 4**), whereas PC1’ was positively correlated with IL-1β (P = 0.006, r = 0.6187) (**Figure 4**).

### Correlations between neutrophils, leukocytes, and lymphocytes and plasma cytokines

Correlation analysis of plasma cytokines revealed that IFN-γ was negatively correlated with neutrophils (P = 0.0404, r = -0.2970) (**Figure 4**). IL-1β was positively correlated with leukocytes (P = 0.0031, r = 0.4266) (**Figure 4**), and IL-8 was positively correlated with lymphocytes (P = 0.0074, r = 0.3901) (**Figure 4**). No significant correlations were found between the other cytokines and neutrophils, leukocytes, or lymphocytes (P > 0.05).

## Discussion

*Cryptococcus neoformans*, designated the top fungal pathogen on the WHO’s Fungal Priority Pathogens List in October 2022, is a common environmental fungus that poses a severe risk for immunocompromised individuals, particularly those with HIV, as it leads to cryptococcosis–an infection with high morbidity and mortality rates among this population. Although the global incidence of HCM is decreasing, the case fatality rate remains alarmingly high, often exceeding 50% outside of controlled clinical settings. This high mortality rate is particularly notable in low- and middle-income countries (Tugume et al., 2023).

*Cryptococcus* typically spreads through the respiratory tract, where an initial immune response in the lungs can eradicate or suppress the fungus. In immunocompromised patients, however, the pathogen disseminates, leading to fungemia and eventually CNS invasion. Within the immune-privileged CNS, *Cryptococcus* employs mechanisms such as the “Trojan horse” and paracellular/transcellular routes to breach the blood–brain barrier, aided by virulence factors such as a polysaccharide capsule, melanin, and laccase (Tugume et al., 2023; Zhao et al., 2023). This study explored immune dynamics in AIDS patients with cryptococcal infection, focusing on Th1/Th2 cytokines, over a 28-day treatment period. Cytokines play a recognized role in HCM immunopathogenesis, with an imbalanced Th1/Th2 response linked to worse disease outcomes.

In this study, the fungal cultures of 23 patients and 16 patients were confirmed. Although fungal culture is the gold standard for confirmed diagnosis, its sensitivity is limited by multiple factors, such as improper sample transport or storage conditions. In resource-limited areas, especially in Africa and Southeast Asia, microscopic examination via ink staining is the first-line method for diagnosing cryptococcal meningitis. In patients with HIV-associated cryptococcal meningitis, the CSF usually has an extremely high fungal load (>10^5^ CFU/mL), which makes the sensitivity of ink stain microscopy close to 100%. The rate of missed detection of *Cryptococcus* in culture is approximately 30%, as reported by Rajasingham et al (Rajasingham et al., 2017). In the present study, 100% of the CSF ink-stained samples were CSF smears. However, the positive rate of CSF culture was 69.57%, which is consistent with previous studies, further suggesting that cultures may cause false negatives due to sample processing delays, etc. Considering the above reasons, all 23 patients included in this study were considered to have a confirmed diagnosis of HIV-related cryptococcal meningitis. The clinical features of our HCM patients and the reduction in CD4^+^ T cells are consistent with previous reports. However, the deceased group had a significantly greater average age and lower cerebrospinal fluid opening pressure than did the survivor group. A previous study identified advanced age, low CD4^+^ counts, underweight, low CSF pressure, and anemia as predictors of mortality in HCM patients (Jarvis et al., 2014). Our findings also revealed that neutrophil counts were lower in the deceased group, with many patients lacking a significant inflammatory response, which may facilitate cryptococcal spread. Neutrophils, which are essential to innate immunity, can be suppressed by the cryptococcal xylomannanoguronide capsule (Rocha et al., 2015), and the inhibition of neutrophil extracellular traps further aids infection dissemination and progression, serving as an additional immune evasion mechanism. Furthermore, a CD4 count <100 cells/µL is a major risk factor for the morbidity and mortality of cryptococcal meningitis. Globally, 70% of patients have CD4 <100 cells/µL, and mortality is negatively correlated with CD4 levels. CD4 <50 cells/µL and 28-day mortality in patients with CD4 <50 cells/µL can reach 40%-60% without timely antifungal therapy and delayed initiation of ART. The median CD4 level was lower in Africa (e.g., South African median CD4 = 27 cells/µL), whereas patients in high-income countries had a slightly higher median CD4 level (CD4 = 50–100 cells/µL); however, differences in prognosis may be related to treatment accessibility. A low CD4 count weakens the host’s ability to clear *cryptococcus*, which may lead to an elevated fungal load and uncontrolled inflammation (Rajasingham et al., 2017). The proportion of patients with a poor prognosis in the CD4 <50 cells/µL group (14.3%) was lower than that in the CD4 50–200 cells/µL group (28.6%), and this difference may be related to the small sample size or confounding factors (timing of ART treatment). Notably, no poor prognosis was observed in the CD4 >200 cells/µL group, which is in line with the global trend and suggests that improved immune status may reduce risk. Moreover, the results of this study suggest that even with a CD4 concentration >50 cells/µL, we should still be vigilant against cryptococcal meningitis, especially when neurological symptoms exist.

Our key findings of ten cytokines/chemokines in the cerebrospinal fluid and plasma of 23 HCM patients include (1) significant decreases in the IL-2 and IL-10 levels in the cerebrospinal fluid by day 28 of antiretroviral and antifungal therapy; (2) similar decreases in the IL-10 and IL-4 levels, whereas the TNF-α level remained stable between days 1 and 14 before increasing on day 21; and (3) a negative correlation between the IFN-γ level and the neutrophil count, while the IL-1β level was positively correlated with the leukocyte count and the IL-8 level with the lymphocyte count over a 28-day period. The role of cytokines in the immunopathogenesis of HCM is well established (Boulware et al., 2010; Jarvis et al., 2015; Mora et al., 2015). Th1-associated cytokines, such as IL-1β, IL-2, IFN-γ, and TNF-α, play crucial roles in macrophage recruitment, activation, and the generation of reactive oxygen species to combat fungal infections while promoting CD4^+^ T-cell responses. However, their protective versus pathogenic roles in *Cryptococcus* infections continue to be explored (Sabiiti and May, 2012; Wang et al., 2015). On the other hand, Th2 cytokines suppress cellular immune reactions, inhibit T-cell proliferation, and are associated with poor infection control and unfavorable outcomes (Murdock et al., 2014; Jarvis et al., 2015). Xu et al. reported elevated levels of both Th1 and Th2 cytokines in HIV^+^ CM^+^ patients compared with those in HIV^-^ CM^-^ patients, indicating a complex interplay between the Th1–Th2 balance during infection (Xu et al., 2019). Similarly, Li et al. reported decreased levels of IL-2, IL-13, IL-17A, and other markers in 12 HCM patients following antifungal therapy, which aligns with our findings of a decline in IL-4 and IL-10 over 28 days. The observed decreases in IL-4 and IL-10 during antifungal treatment may reflect both fungal clearance and a gradual restoration of Th1/Th2 immune balance. IL-10, an anti-inflammatory cytokine that suppresses macrophage activation and Th1 responses, is often elevated in advanced HCM and correlates with poor fungal clearance. Therefore, its decline over 28 days suggests attenuation of the immunosuppressive milieu as fungal burden decreases. Similarly, the reduction of IL-4, a Th2 cytokine promoting humoral rather than cellular immunity, may indicate a shift away from Th2 dominance toward improved cell-mediated antifungal defense.

The balance between proinflammatory and anti-inflammatory signals is crucial in managing fungal infections. In our study, where all infections were confirmed as *Cryptococcus neoformans* by mNGS, this balance was particularly evident. Research indicates that *C. neoformans*, when compared to *C. gattii*, has a greater tendency to induce a Th2-biased immune response, which is generally associated with disease progression. This may explain the significant baseline levels of the Th2 cytokines IL-4 and IL-10 observed in our patients. Consequently, the significant decrease in both IL-10 (in plasma and CSF) and IL-4 (in plasma) following treatment is a key finding. It suggests that effective antifungal therapy, by reducing the fungal burden, successfully alleviates the pathogen-driven Th2-skewing, which is critical for controlling the infection.

An effective immune response to *Cryptococcus* involves coordination among Th1, Th2, and Th17 cells to control fungal proliferation while minimizing tissue damage (Medzhitov et al., 2012; Rohatgi and Pirofski, 2015; Gorenec et al., 2016). This immune shift was further characterized by the dynamics of Th1 cytokines. In HIV-infected individuals, infection often reduces Th1 cells and increases Th2 cells, complicating the immune response to brain cryptococcosis (Klein et al., 1997; Jiang et al., 2011). The Th1 cytokine TNF-α remained stable from days 1–14 and increased by day 21, likely reflecting a restored capacity for a macrophage-led response, while the pro-inflammatory cytokine IL-2 concurrently decreased in the CSF, signaling a resolution of acute inflammation as the infection was controlled. This complex response is especially relevant in HIV-infected individuals, where the underlying disease often reduces Th1 cells and increases Th2 cells, complicating the immune response to brain cryptococcosis. Our findings of reduced IL-10 in plasma and CSF align with those of MoL et al., although Okafor et al. reported no significant correlations for most analytes, except for weak negative associations for IFN-γ and IL-4 during early infection (Gorenec et al., 2016).

Interferon-γ and neutrophils are integral to the immune defense against *Cryptococcus neoformans (*Arora et al., 2005; Hardison et al., 2010). Joseph N. Jarvis et al. demonstrated that combining traditional therapy with short-course IFN-γ significantly improved cryptococcal clearance in CSF (Jarvis et al., 2012). Similarly, a positive correlation between neutrophils and IFN-γ was reported by Dong et al. during a 41-day study (Dong et al., 2019), further underscoring the importance of this cytokine and neutrophils in immune modulation. However, our findings revealed a negative correlation between IFN-γ and neutrophils (P = 0.04, r = -0.297), diverging from these reports. Further exploration of the role of neutrophils in inflammation revealed that IFN-γ may have a dual role in HIV-associated cryptococcal meningitis: it both participates in antifungal immunity and aggravates immunodeficiency by inhibiting neutrophils. Schroder et al. reported that IFN-γ promotes the generation of monocytes during inflammation while inhibiting the development of neutrophils, which may lead to reduced numbers of neutrophils (Schroder et al., 2004). Hu et al. reported that IFN-γ-pretreated macrophages inhibit part of the inflammatory response program and reduce the recruitment of neutrophils (Hu et al., 2008).

Thus, there is a similar case. Notably, although HIV infection causes decreased overall IFN-γ levels, interindividual differences (e.g., some patients retain strong Th1 response capacity) may lead to more significant neutrophil depletion in patients with relatively high IFN-γ levels, thus driving the negative correlation. Such a regulatory pattern may represent an adaptive immune mechanism that limits excessive inflammatory injury while maintaining sufficient fungal clearance, reflecting a delicate balance between host protection and immunopathology during antifungal therapy.

This study revealed a significant positive correlation between plasma IL-1β levels and white blood cell count (P = 0.0031, r = 0.4266). These results suggest that IL-1β may play an important role in the inflammatory response in cryptococcal meningitis. IL-1β is a key proinflammatory cytokine known to be involved in the regulation of immune responses and the release of inflammatory mediators in a variety of inflammatory diseases. In their review, Dinarello et al. clearly indicated that IL-1β can increase cortisol secretion through the hypothalamus–pituitary–adrenal axis, transiently inhibiting lymphocytes while promoting the release of neutrophils from the bone marrow. This finding is consistent with the fact that IL-1β levels were positively associated with leukocyte count in the context of systemic inflammation triggered by cryptococcal infection in this study (Dinarello, 2009). In addition, Michael M Lederman et al. reported that sustained immune activation (e.g., increased IL-1β and IL-6 levels) was associated with increased leukocyte subsets, especially monocytes and neutrophils, in HIV-infected patients receiving antiviral therapy. Cryptococcal meningitis, which is an opportunistic infection, may exacerbate this inflammatory state, leading to IL-1β-driven leukocyte mobilization (Lederman et al., 2011).

IL-8 (CXCL8) is generally recognized as a key chemokine for neutrophils, and its plasma level is positively correlated with neutrophil infiltration. However, the present study revealed a significant positive association between plasma IL-8 levels and lymphocyte counts without a significant association with neutrophil counts in patients with HIV-associated cryptococcal meningitis. This difference may be related to the unique immune microenvironment of HIV infection, and IL-8 may recruit activated lymphocytes through CXCR1/CXCR2 signaling in advanced AIDS patients (Baggiolini et al., 1989; Lane et al., 1999; Kedzierska et al., 2003). Since the trend of CSF chemokine levels versus neutrophils was not explored in this study, further verification of whether the chemotactic target cells of IL-8 differ by immune background is needed in the future. We speculate that, in patients with HIV-associated cryptococcal meningitis, lymphocytes rather than neutrophils may serve as the major inflammatory cells responsible for fungal clearance.

This study revealed significant differences in cytokine dynamics between the plasma and CSF of HIV patients with cryptococcal meningitis during antifungal therapy via PCA. In plasma, PC1 was dominated by Th2-related factors (IL-4, IL-13) and proinflammatory factors (IL-6, IL-1β) and was inversely associated with IL-12P70 (Th1 driver) and TNF-α, suggesting that Th2 polarization is involved in systemic immunosuppression. However, in the CSF, PC1′ (IL-1β, IL-13, IL-4, and IL-8) coexist with PC2′ (IFN-γ, TNF-α, and IL-2), indicating a mixed Th1/Th2 response within the CNS, which may reflect a competitive mechanism of local immune activation and pathogen clearance.

The Th2 bias of PC1 in plasma (strong positive IL-4 correlation, r = 0.85) may be attributed to CD4^+^ T-cell depletion and chronic immune activation due to HIV infection, thereby inhibiting the Th1-type response (e.g., IL-12P70 negative weight). This finding is consistent with the observations of Jarvis and Harrison that *Cryptococcus* infection is strongly associated with upregulated Th1 deficiency (IFN-γ, IL-12) and Th2/regulatory cytokines (IL-4, IL-10) in HIV patients (Jarvis and Harrison, 2007). Furthermore, cryptococcal capsular polysaccharides may exacerbate the Th1/Th2 imbalance by inhibiting dendritic cell maturation and further reducing IL-12 secretion (consistent with the negative association of IL-12P70 in PC1) (Vecchiarelli et al., 2003). The coexistence of PC1′ (IL-1β, IL-4) and PC2′ (IFN-γ, TNF-α) leads to IL-1β- and IL-8-driven inflammation and local activation of the Th1 response. Among them, the inflammatory response may reflect the microglia and astrocyte activation induced after cryptococcal invasion into the CNS, leading to blood–brain barrier disruption and neutrophil recruitment. The weights of IFN-γ and TNF-α in PC2′ (Th1 markers) may represent a host attempt to control infection by increasing macrophage killing in response to local activation of the Th1 response. However, the coexistence of IL-4 and IL-13 (Th2) may weaken fungal clearance efficiency by inducing alternative macrophage polarization (M2 phenotype), suggesting that Th1/Th2 imbalance may affect the treatment response and prognosis. Moreover, the significant contribution of IFN-γ and TNF-α in CSF PC2’, together with the observation that CSF IL-10 levels declined markedly only at day 14 of antifungal therapy, whereas plasma IL-10 levels decreased significantly by day 7, supports the hypothesis of “tissue-specific immune heterogeneity in fungal infections” proposed by Romani, suggests that the CNS microenvironment may be partially resistant to systemic immunosuppression (Romani, 2011).

During antifungal therapy, the gradual reduction of IL-10 and IL-8 likely reflects decreased fungal burden and consequent alleviation of pathogen-driven immune activation. In contrast, the transient increase of TNF-α observed in some patients could represent immune rebound rather than drug toxicity. As fungal antigens are released during rapid killing, immune reactivation may occur locally, leading to elevated TNF-α and IFN-γ as markers of macrophage activation. This pattern resembles a mild, localized immune reconstitution phenomenon, but notably, no patients in our cohort met the diagnostic criteria for IRIS, suggesting that this immune rebound was controlled and beneficial for fungal clearance.

Systemic Th2 polarization and local Th1 response retention in the CNS confirmed the “Th1 protective immunity” theory proposed by Kawakami et al., indicating that the Th1 immune response (marked by IFN-γ, IL-12, and TNF-α) plays a key role in the control of cryptococcal infection, whereas the Th2 response (marked by IL-4 and IL-13) may aggravate infection by inhibiting macrophage activity (Kawakami et al., 1996). This study revealed that the immune response of HIV patients with cryptococcal meningitis presented the dual feature of “peripheral Th2 polarization–local Th1/Th2 competition in the CNS”, which may provide a target for individualized immunomodulatory therapy. Further validation of the clinical benefits of Th1 augmentation strategies (e.g., IFN-γ) or Th2 inhibition therapy in such patients is needed in the future.

This study highlights novel aspects of immunological characteristics during cryptococcal infection and antifungal therapy but is not without limitations. First, only 23 patients were included in the study. The small sample size may reduce the statistical power and thus limit the universality of the results. Second, as a retrospective analysis of clinical cases, our study lacked control groups. All patients received standard-of-care antifungal therapy immediately upon diagnosis in accordance with ethical and clinical guidelines, making an untreated cohort unavailable for comparison. Similarly, for practical reasons in a clinical setting, comprehensive immunological panels were not systematically performed on all patients prior to the initiation of therapy.

Next, the follow-up period was short. A 28-day observation period cannot assess long-term immune recovery or a delayed inflammatory response (e.g., immune reconstitution syndrome). The dynamic trend of some cytokines (e.g., TNF-α is significantly increased at 21 days) may require longer observations to clarify their clinical significance; additionally, the detection method and scope have limitations. We measured only a fraction of cytokines, and other key mediators (e.g., GM-CSF) were not covered. Furthermore, our analysis was limited by the lack of data on fungal burden. Due to institutional protocols prioritizing rapid diagnosis via mNGS, quantitative measures such as cryptococcal antigen (CrAg) titers or colony-forming units (CFU/mL) were not available. This prevented a direct correlation between cytokine dynamics and the rate of fungal clearance. Only a fraction of cytokines and other key media (e.g., GM-CSF) are not covered. Information about important immune pathways may be lacking.

Last, mechanistic studies that describe only the correlation, not the causal mechanism by *in vitro* experiments or animal models, which are unable to define the function of the cells, are lacking. Despite these limitations, our findings provide valuable insights into the host immune response against fungal infections. This study provides a foundation for future investigations into the immunological mechanisms of cryptococcal meningitis, ultimately aiding in the development of improved therapeutic strategies.

## Data Availability

The original contributions presented in the study are included in the article/**Supplementary Material**. Further inquiries can be directed to the corresponding authors.
