# Supplementary material for: Cytokine dynamics in the blood and cerebrospinal fluid of HIV/AIDS patients with cryptococcal meningitis receiving antifungal therapy
Source: Front Cell Infect Microbiol. 2025 Dec 5;15:1691025. doi: 10.3389/fcimb.2025.1691025 (PMC12715003; doi:10.3389/fcimb.2025.1691025)

**Table S1. Comparison of Demographic and Clinical Features between Surviving and Deceased Patients with HIV-Associated Cryptococcal Meningitis**

|  | Total case(n=23) | | p |
| --- | --- | --- | --- |
|  | Survival(n=19，82.6%) | Death（n=4，17.4%）) |  |
| **Age** (years) | 45.26±10.91 | 62.25±13.12 | .012* |
| Gender |  |  | .999 |
| Male | 15（83.3%） | 3（16.7%） |  |
| Female | 4（80%） | 1（20%） |  |
| Occupation |  |  | .570 |
| Unemployed | 11(73.3%) | 4(26.7%) |  |
| Farmer | 5(100%) | 0 |  |
| Worker | 3(100%) | 0 |  |
| Matrimony |  |  | .999 |
| Married | 13（81.3%） | 3（18.8%） |  |
| Divorce | 3（75%） | 3（25%） |  |
| Unmarried | 3（100%） | 0 |  |
| HIV transmission route |  |  | .642 |
| Sexual transmission | 6（66.7%） | 3（33.3%） |  |
| Intravenous drug use | 4（100%） | 0 |  |
| tattoo | 2（100%） | 0 |  |
| Unknow | 7（87.5%） | 1（12.5%） |  |
| ART before hospitalization |  |  | .999 |
| Yes | 4(80%) | 1(20%) |  |
| No | 15(83.3%) | 3(16.7%) |  |
| Clinical symptoms |  |  |  |
| Fever | 10(76.9%) | 3(23.1%) | .604 |
| Dizziness and headache | 15(88.2%) | 2(11.8%) | .270 |
| Cough and sputum | 6(75%) | 2(25%) | .490 |
| Nausea and vomiting | 6(85.7%) | 1(14.3%) | .792 |
| Lose weight | 7(87.5%) | 1(12.5%) | .644 |
| Neurological symptoms and signs |  |  |  |
| Disturbance of consciousness | 7(100%） | 0 | .273 |
| Convulsion | 2(66.7%) | 1(33.1%) | .453 |
| Blurred vision | 8(100%) | 0 | .257 |
| Psychobehavioral abnormality | 2(100%) | 0 | .999 |
| Swoon | 1(50%) | 1(50%) | .324 |
| Decreased muscle strength | 2(66.7%) | 1(33.1%) | .453 |
| Memory decline | 4(100%) | 0 | .999 |
| Meningeal irritation sign | 13(92.9%) | 1(7.1%) | .260 |
| Pathological sign | 6(100%) | 0 | .539 |
| **Intracranial pressure** |  |  | .038* |
| >200 mmHg | 16(88.9%) | 2(11.1%) |  |
| 200 mmHg～100 mmHg | 3(100%） | 0 |  |
| ≦100 mmHg | 0 | 2(100%) |  |
| Concomitant disease |  |  |  |
| Pulmonary infection | 16(80%) | 4(20%) | .999 |
| thrush | 7(70%) | 3(30%) | .281 |
| Liver dysfunction | 8(72.7%) | 3(27.3%) | .317 |
| Renal insufficiency | 4(80%) | 1(20%) | .999 |
| Diseases of blood system | 10(76.9%) | 3(23.1%) | .604 |
| Hypokalemia | 9(81.8%) | 2(18.2%) | .999 |
| Malnutrition | 10(83.3%) | 2(16.7%) | .999 |
| Laboratory examination |  |  |  |
| CSF turbid | 2(100%) | 0 | .999 |
| CSF globulin positive | 16(80%) | 4(20%) | .999 |
| CSF leukocyte(*10^6/L) | 29(110,1~260) | 49(383.5,9~517) | .557 |
| CSF chlorine(mmol/L) | 123.8(8.8,104.4~132.8) | 120.45(20.9,109.5~136.6) | .667 |
| CSF glucose(mmol/L) | 1.73(1.43,0.11~3.93) | 2.09(1.46,0.68~2.54) | .557 |
| CSF albumin(mg/L) | 739(813.8,235.8~1610.2) | 696.2(1262.8,503.5~2120.7) | .845 |
| Leukocyte in blood(*10^9/l) | 5.28（2.92，1.35～15.46） | 3.22（3.03，1.25～4.46） | .081 |
| **Neutrophils in blood** (*10^9/l) | 3.94（1.65，0.72～12.94） | 2.18（1.34，1.28～2.79） | .033* |
| Lymphocyte in blood(*10^9/l) | 0.54（0.47，0.21～3.91） | 0.82（1.48，0.35～2.03） | .837 |
| Monocyte in blood(*10^9/l) | 0.42（0.47，0.09～0.8） | 0.3（0.3，0.19～0.52） | .534 |
| Hemoglobin(g/l) | 127（39，108～176） | 104（43，98～151） | .172 |
| Patelet(*10^9/l) | 128（125，29～276） | 85.5（129.25，28～194） | .227 |
| CD4(/ul) | 34.1(34.94,5.62~211) | 55.91(70.1,25.6~115.46) | .227 |
| CD8(/ul) | 344.5(274.9,134~3614) | 777(1289.58,169~1565.77) | .538 |
| CD4/CD8 | 0.19(5.94,0.01~15) | 0.08(0.25,0.04~0.36) | .386 |
| Serum amyloid A(mg/l) | 35.95(131.6,0.7~263.7) | 49.5(0.6~196.3) | .842 |
| Hypersensitive C protein(mg/l) | 5.92(17.79,0.53~58.74) | 13.16(69.20,0.33~84.6) | .902 |
| L-6(pg/ml) | 17.51(18.7,1.5~60.73) | 17.78(1.17~32.33) | .740 |
| Procalcitonin(ng/ml) | 0.076(0.11,0.02~0.34) | 0.41(1.54,0.06~1.95) | .098 |
| Na(mmol/L) | 133.25(3.08,127~135.8) | 132.1(5.42,127.3~134) | .357 |
| K(mmol/L) | 3.92(0.91,2.61~4.82) | 3.62(0.69,3.36~4.24) | .999 |
| ALT(U/L) | 16(53.75,8~158) | 20(39.9,5.8~51) | .412 |
| AST(U/L) | 15.5(36.75,11~83) | 36(51.75,9~66) | .571 |
| RNA | 2.44E+5(2.9E+6,100~6.47E+6) | 8.38E+5(5.78E+6,4.45E+5~8E+6) | .250 |
| Medicate |  |  | .247 |
| Amphotericin B+Fluconazole+Fluorocytosine | 10(90.9%) | 1(9.1%) |  |
| Amphotericin B+Fluconazole | 7(77.8%) | 2(22.2%) |  |
| AmphotericinB+Fluorocytosine | 2(100%) | 0 |  |
| Amphotericin B+Fluiconazole | 0 | 1(100%) |  |

*Statistically significant.

**FigureS1. Flow chart of** **sample attrition**


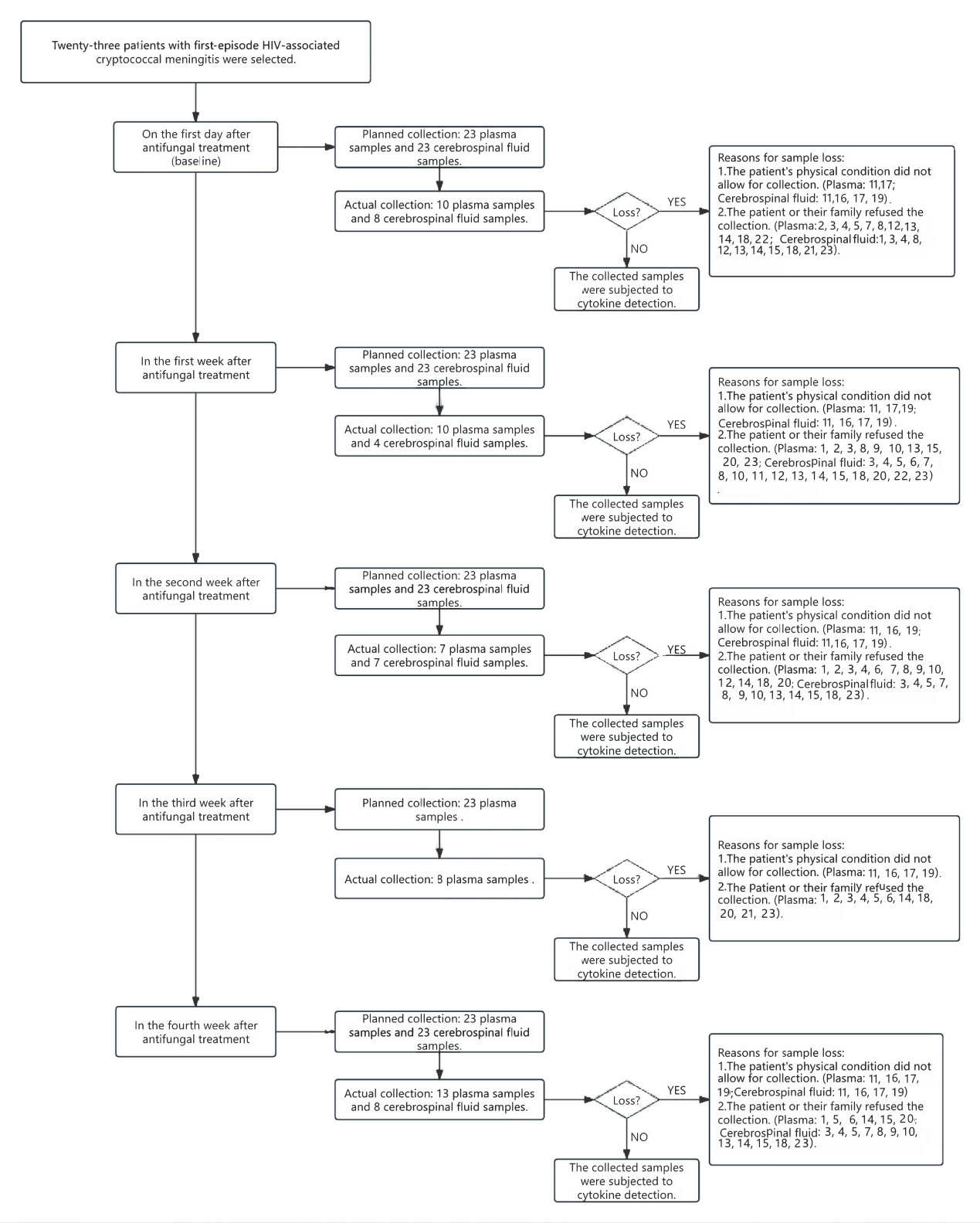

Supplement: Supplementary file 1 [file Table1.docx]
